# An Exploratory Study of ICU Pediatric Nurses’ Feelings and Coping Strategies after Experiencing Children Death

**DOI:** 10.3390/healthcare11101460

**Published:** 2023-05-17

**Authors:** Mirian Anguis Carreño, Ana Marín Yago, Juan Jurado Bellón, Manuel Baeza-Mirete, Gloria María Muñoz-Rubio, Andrés Rojo Rojo

**Affiliations:** 1 Cruz Roja Española, Asamblea Cartagena, 30202 Murcia, Spain; 2 Pediatric Intensive Care Unit, Hospital Universitario Virgen de la Arrixaca, Public Murcian Healthcare System, 30120 Murcia, Spain; 3 Faculty of Nursing, Catholic University of Murcia (UCAM), 30107 Murcia, Spain; 4 Intensive Care Unit, Hospital Universitario Virgen de la Arrixaca, Public Murcian Health System, 30120 Murcia, Spain

**Keywords:** intensive care units, pediatric, pediatric nurse, psychological adaptation, death experiences, qualitative study

## Abstract

Background: This study aims to explore the feelings and experiences of nursing staff when faced with the death of a pediatric patient in the ICU. Methodology: A qualitative study based on hermeneutic phenomenology was conducted through semi-structured interviews. Ten nurses (30% of staff) from the Pediatric Intensive Care Unit of a referral hospital were interviewed in April 2022. Text transcripts were analysed using latent content analysis. Results: Content analysis indicated that the interviewees had feelings of sadness and grief; they had a misconception of empathy. They had no structured coping strategies, and those they practiced were learned through personal experience, not by specific training; they reported coping strategies such as peer support, physical exercise, or strengthening ties with close family members, especially their children. The lack of skills to cope with the death and the absence of support from personnel management departments were acknowledged. This can lead to the presence of compassion fatigue. Conclusions: The feelings that PICU nurses have when a child they care for die are negative feelings and sadness, and they possess coping strategies focused on emotions learned from their own experience and without institutional training support. This situation should not be underestimated as they are a source of compassion fatigue and burnout.

## 1. Introduction

Pediatric death is a process that affects everyone who is close to it or close to the child. Its biological, psychological, social, and spiritual effects are felt by families and healthcare professionals. The death of a child in the Pediatric Intensive Care Unit (PICU) is a rare event that can take many forms, usually following a decision to limit life-sustaining treatment (LoLST) [1,2].

The overall mortality rate in the Pediatric Intensive Care Unit (PICU) is lower than in the adult Intensive Care Unit (ICU). In Spain, in the last 15 years, between 2 and 4% of patients admitted to type III units died within 24 h of admission after failed CPR.

These deaths affected the under-one-year age group more than the other age groups (approx. 30%), probably due to perinatal causes such as malformations, cardiac arrest, or peripartum hypoxia. Deaths occurred more frequently in the afternoon–night shift (62%) after an average stay in the PICU of 3 days (1–12 d) [1,2].

The death of children older than one year in PICU is because they are children who have overcome periods of critical illness since birth, and this has become chronic. The death of these children may present psychological harm to healthcare professionals, due to the connotations and emotional bonds established in previous admissions.

Both the figures for pediatric mortality due to acute illness and LoLST in Spanish PICUs are similar to those registered in PICU health settings in both Europe and North America [3,4].

Unlike what occurs in adult patients, the characteristics of patients who die in PICU correspond to two profiles: (a) patients under 1 year of age, with acute health problems related to short-stay perinatal problems (less than 2 days); or (b) patients with chronic diseases, who are admitted to PICU after an exacerbation of their pathology or failure in their chronic treatment. In both cases, LoLST is mostly applied [5].

Given that the presence of families is constant (unlike what happens in the critically ill patient), it is possible to speak of “family care” rather than exclusively child care. Family presence at the bedside of critically ill children is recommended as part of ‘family-centred care’. Other research reports that parents report anxiety, fear, conflict, and information overload [5], along with feelings of isolation from the outside world and moments of collapse. They feel that they have lost control and feel alone [6].

That is why the health professionals who work in these units must be familiar with the LoLST process, and with the ambivalence that it implies, working with the death of a child without suffering and the “unnatural” at a psychological level about the emotional death of a child. Training in these processes is essential, especially in handling situations of family grief.

The impact of the death of a child on the family is well-known, as well as the impact of health professionals’ actions on the family in the context of the death of a child [6] and the use of communication strategies by health professionals to support the family’s coping with the impact of the death of a child [7,8].

The nurses that experience a death of a patient see an impact in their working and social life, manifesting as anxiety and emotional exhaustion [9]. Some research had found clinical nurses who related overwhelming psychological stress and negative attitudes toward death due to their experiences with patient death, and the majority of them felt unprepared [10,11,12]. The way professionals face death and suffering depends on their abilities and personal resources, as the same stressful event generates very diverse reactions in each individual [10]. These behaviors are known at the level of therapeutic psychology as coping strategies (CS). CS are often defined as “constantly changing cognitive and behavioural efforts to manage specific external and/or internal demands that are appraised as taxing or exceeding the resources of the person” [13]. They are ways of thinking and behaving, adopted to reduce the difficulty experienced in a given situation, whether this difficulty is internal or external [14].

Human coping strategies in the face of negative or stressful situations are diverse. For this reason, no two people respond in the same way to a given situation. As Lazarus and Folkman’s Cognitive Motivational Relational Theory (CMRT) [15] indicates, the cognitive component is essential in this differentiation; the mental evaluation of the situation and the availability of coping resources are what conditions the human response to adversity. In this sense, identifying the mental framework understood as the attitudes that the person possesses to cope with a situation conditions that person’s response to negative events.

The objective of this study is to describe and understand the experiences, perceptions, and coping strategies of nurses who have suffered a pediatric death in ICUP.

## 2. Materials and Methods

### 2.1. Study Design

The study was performed from a constructivist point of view using Van Manen’s hermeneutic phenomenology [16]: the researcher is primarily interested in the study of the essential meaning of phenomena as well as the meaning and importance they have. In addition, it is necessary to emphasize that, from this approach, “problems to be solved” are not raised, but rather questions about the meaning and meaning of a certain experience.

To obtain the deep experience of the participants, this study adopted qualitative exploratory research to collect data in the form of a semi-structured interview.

The word “hermeneutics” etymologically refers to the art of expressing or interpreting an action, an expression, or a text, performed or expressed by another person and to which we are witnesses. His intention, therefore, is to look beyond the literal meaning of the words to find, perhaps, something more profound [17].

The development of the study followed the phases of a Gadamerian-based research method: to understand the participants’ stories, we must be predisposed to be told something, identify our prejudices, know the reality they tell us, and then analyze the text and question what they are trying to tell us so that meanings emerge [18].

The conducting and reporting of this study were guided by the Standards for Reporting Qualitative Research (SRQR) [19].

### 2.2. Researchers Characteristic

The first author conducted all interviews. Her own nurse undergraduate training took place between 2014 and 2018. She was not known to the participants of this research prior to undertaking the study and deliberately did not undertake any clinical or teaching activities locally alongside this research. She knew the environment and the reality of intensive care of the pediatric patient during her clinical training in the last year of her nursing degree education. The rest of the researchers have first-hand knowledge of the reality of pediatric critical care.

### 2.3. Context: Setting/Site and Salient Contextual Factors

The study was conducted in a III-level PICU (where all types of emerging pathologies of pediatric patients are treated: cranioencephalic trauma, cardiac surgery, respiratory problems, renal care, etc.) belonging to a public hospital in the southeast of Spain for a population of approximately 1.5 million inhabitants, with 13 multipurpose beds, in which children with critical pathologies are treated from 21 days of life (neonates in the case of cardiac surgery) up to 14 years of age. The unit is staffed by a total of 25 nurses specialized in pediatric care, 5 registered nurses in training as pediatric care specialists, 10 healthcare assistants, and 8 pediatricians specialized in critical care.

Characteristic of the study population, in the Spanish National Health System, since 2010, there has been a voluntary and binding specialization of nursing professionals in various clinical areas. Specialization as a pediatric nurse is one of them. The specialization process is carried out in the form of nurse intern resident: during a period of two years, theoretical training is carried out accompanied by specialized stays in child care services. This means that, at present, the vast majority of professionals working in PICUs in the country are specialist pediatric nurses, which gives them a special knowledge of the physiopathology of the child, as well as not being “contaminated” with adult care. The child is not a “miniature adult”.

### 2.4. Sample

The sample consisted of nurses belonging to the related PICU. Participants were recruited by snowball convenience sampling and key informant figure. A total of 10 participants were interviewed. It represents 30% of the study population. Inclusion criteria were: (1) nurses who had directly cared for a critically ill child who died in PICU; (2) nurses with at least one year of experience in PICU; and (3) nurses who were able to speak clearly and were willing to participate in the research. Nurses in training as child care specialists were included.

None of the nurses interviewed showed signs of personal or professional trauma that prevented them from being interviewed.

The participants were mostly female nurses (*n* = 9) aged between 22 and 47 years (median age 36; Q1 23; Q3 43 years), with a nursing experience between 2 and 25 years (median age 14; Q1 6; Q3 22 years); nine of the participants were married and one was single. The median pediatric ICU experience was 13 years. Thirty percent of the participants had experience in other special services such as adult ICU before working in pediatric ICU. The basic information of the participants is shown in Table 1.

### 2.5. Data Collection Methods

A semi-structured interview was conducted. Ten interviews were conducted, one for each participant. The interview guideline was drawn up based on the purpose of the research and the literature review and included the following: (1) Can you talk about your experience when a child you were caring for died? (2) Regarding the death of a child, can you share your experience and feelings about it? (3) Do you have negative feelings related to the death of a child you cared for, and can you tell us about those feelings? (4) Can you tell us about the actions you take to combat negative feelings after the death of a child you cared for? (5) Do you know of any emotional support programmes for nurses who experience feelings like yours after the death of a child you were caring for? (6) Concerning the experience you have had following the death of a child you were caring for, do you have any other things would like to share with me?

The interviews were conducted in the work environment itself, during the professionals’ working day, using time slots with a low workload in an environment with adequate privacy and sound conditions. The interviews took 35 min on average.

The research team contacted the subjects proposed for research and set a suitable date and time for the interview.

The same interviewer (MAC) was present in all interviews; no other person was present during the interview. The session began with a welcome to the participant, followed by the reading and signing of the informed consent form. The interview began with opening questions about the work of the participants, followed by the core issues.

The interviews were audio-recorded and transcribed by the research team. The transcriptions were complemented with field notes taken by the interviewer. Two members of the study team (AMY and CPV) independently coded each transcript using conventional content analysis methods as their methodological orientation. Both team members continued to code independently and met to review the coding by consensus after every 5 additional transcripts.

Neither the transcripts nor the codes were reviewed by the interviewees.

Data collection took place between 1 April and 30 April of 2022.

### 2.6. Data Analysis

Audio records were transcribed verbatim and cross-checked by the research team.

The interviews were recorded using a recording device and transcribed verbatim. Analysis was carried out using latent content analysis [20]. Within the coding process, open coding was performed.

To develop codes, two researchers conducted several independent readings of the transcribed text. This process allowed researchers to familiarize themselves with the scope of the data, stimulating inchoate ideas about possible concepts or constructs that may exist in the data. A priori codes were generated, based on the topics of the questions, such as reactions to the child’s death and behaviours after the death. Other codes that emerged during the analytical process (emergent codes) were added to the a priori codes that were identified before the analysis began.

Each researcher coded the texts independently. After the initial coding, joint sessions were held to assess the degree of agreement of the researchers in each case. Where discrepancies occurred, a senior researcher was involved. After discussing the differences found, the codes are consolidated to create categories. Categories are relationships between codes that represent a higher level of abstraction in the data. To draw conclusions and interpret the fundamental meaning underlying the data, categories are organized into themes.

### 2.7. Research Rigidity

To ensure rigour and credibility, this study followed the standards proposed by Lincoln and Guba [21]. Reliability was ensured by describing the data analysis in detail and providing direct references to reveal the basis on which the analysis was conducted. The researchers coded the interviews independently of each other. Consistency of analysis was determined by a meeting to discuss initial findings, where norms and emerging themes were discussed until consensus was reached. Consensus was maintained throughout the coding process.

Given the exploratory nature of our study, as well as the homogeneous sample and the redundant information, the ten interviews yielded enough to achieve data saturation, according to Hennink et al. [22]’s data saturation parameters.

### 2.8. Ethics Approval and Consent to Participate

The study was approved by the Ethics Committee of the Catholic University of Murcia (UCAM) (IRB CE06211).

In this study, all the methods were carried out in accordance with the Declaration of Helsinki and approved by the Ethics Committee of Catholic University of Murcia (UCAM), Spain. All the participants signed written informed consent forms and were voluntarily enrolled in the study. Additionally, they could withdraw from the study at any time. All the participants were assured that their information would remain confidential. No participants withdrew from the study.

To maintain participant confidentiality, participants were coded by interview order, with an alphanumeric code identifying category and order. No other data are recorded on the recordings.

## 3. Results

This study mainly explored the feelings, perceptions, and experiences of nurses who had experience with the death of children in the pediatric ICU, in order to identify ways of personal coping with this situation. In total, the researchers interviewed 10 selected participants. No participant refused to participate or dropped out of the interview process.

From the analysis of the data, the researchers summarized the research findings into four main themes: (a) negative feelings about coping with the death of a child in the ICU; (b) wrong care models: sympathy instead of empathy; (c) coping strategies in the face of the death of the child admitted to the ICU; and (d) lack of training and institutional support.

### 3.1. Negative Feelings about Coping with the Death of a Child in the ICU

This was one of the aims of this research, identify the feelings: identifying feelings about a child’s death. When caring for children who die in the ICU, feelings of helplessness, grief, and frustration arise from not having contributed to saving the life of the child they cared for. On the other hand, feelings of fear, helplessness, ignorance, and grief arise when caring for parents. From the interview data, the researchers divided this stage into “grief, frustration, and sadness over the death of the child” and “fear and helplessness towards the parents”.

#### 3.1.1. Grief, Frustration, and Sadness over the Death of the Child

The death of a child is experienced by nursing staff tragically and negatively. There are no cases where death is understood as something positive. All the manifestations are of professional failure and negative feelings on a personal level. This type of feeling extends to the whole unit, from the pediatric doctors to the auxiliary staff.

*“... a failure, in general... then in particular it can be each case different... but death is a failure here, it is taken as a failure.”*

*E4P2L1*

*“... well, for me... a feeling of impotence for not being able to do more... here you do your utmost to save that child... you try to do everything possible... and sometimes the impossible is also attempted... that is why when the child dies, it is a failure.”*

*E3P1L20*

*“When a child dies, all the time you have invested in him/her is lost... and all your effort, the hopes that you put in that treatment is going to work,... that the venous cannulation that you are doing... and that is costing you a lot of effort and time... is going to contribute to saving the child... and when he passes away... all that effort... that not sleeping next to the child... that stress that you have lived through for the child... has been for nothing. ...”*

*E3P3L2*

*“... it makes you question whether it’s worth all the effort... even if it’s worth studying so much... so that in the end the children die of the things you have studied...” ”... a loss, because of the loss of the child.”*

*E4P3L3*

*“… when an elderly man dies... it is easy to justify himself... because he is a person who has already lived his life... when a child dies, a person dies with his whole life ahead of him.. it is a loss, for society... for his family, because of what has happened to him, in general a loss.”*

*E1P2L12*

*“... you take those feelings home with you... you take them with you; ... it’s inevitable... I mean… you can’t leave those sad feelings here… you hang up your pyjamas, and you’re going to get married, like a new person??? ... No… that’s impossible... many times when we are with a child who is critically ill, or who may die at any moment... we take the children home in your mind… (figuratively…) ... we try not to think about him... but you want to call the colleagues who are working... to know how is he/she... but you make an effort not to do it...”*

*E7P1L11*

This feeling of negativity is transversal, affecting all categories of healthcare staff who work in the unit.

*“… we all feel the death of a child we are treating… the auxiliary staff... the doctors, and us (nurses)... when a child dies, the unit becomes silent... silent... only the sounds are heard noises from monitors and alarms from infusion pumps… until a few hours after death, there is no laughter in the unit... and that we are very smiling and funny...”*

*E7P1L18*

Feelings of doubt appear in relation to whether what has been done has been enough, or if something more could have been done. These doubts affect not only technical skills, but also non-technical ones, in aspects such as communication with the patient and with the family.

*“... the ending would have improved... so that makes me feel that I haven’t done it as well as I could, and that we ourselves are the ones who don’t let things come when healing, that we already know that this doesn’t work... but still… so you think that sometimes you could have done better…”*

*E4P2L12*

*“…there are times when you think you could have done more and I’m not saying it for technical care... but for things related to the family… perhaps the ending would not have changed... he would have died the same... but perhaps because it would have ended in a better way.”*

*E8P2L2*

#### 3.1.2. Fear and Helplessness towards the Parents

Among the surprising stories, we find those that indicate that they have suffered fear and shame in dealing with the family of the deceased children: fear of the reactions that family members have, often anger and frustration that is unleashed towards the health personnel, and that culminates in verbal and/or physical aggression; and shame in the face of the feeling of helplessness for not being able to save their child, and the false feelings of security that have been transmitted, when the child was better. It is a feeling of deception of the family, an unintentional deception.

*“... some parents... have been very upset when it has been said that the child has died,... and… we let them be with them (their child).. and they are seeing that we don’t stop working to save their child... but some parents... you know... they start screaming... they look at us badly... and when they start hitting things and screaming because of the pain... I feel afraid... in case they attack us or something...”*

*E1P1L18*

*“... I have only seen a few aggressive cases... but I understand them... because it is a very hard situation... if that were to happen to me... losing a child... I don’t know how I would react...”*

*E4P2L4*

*“When the child dies and I have to go into the box to do something... I don’t know what to say to the family... especially if I told them a while ago, ‘don’t worry, don’t be afraid... you must be calm…your son are in good hands... and in the best place to recover....’ And when in a while that same family comes in to see their deceased child... well, my face falls with shame and makes me want to cry... because I think that I have generated part of that pain... with false hopes.”*

*E6P1L19*

### 3.2. Wrong Care Models: Sympathy Instead of Empathy

Another of the behaviours detected in the testimonies is the recognition of attitudes of sympathy and affection towards patients and their relatives, which generates a negative feeling when the child dies. However, the concept of “sympathy” is being confused with that of “empathy” by the interviewees.

*“Death is difficult to accept for most people who lose a relative or loved one... and also for the nurse who works with children... because most of the time we end up having a... relationship of empathy... with the parents and the children... very great, because of the time they remain hospitalized and because of the good relationship that is created with the relatives.”*

*E6P2L2*

We understand that the interviewees allude to the concept of an “empathetic relationship with parents and children” when, in reality, it is a concept of sympathy, as there is a treatment of affection, which provokes positive or negative feelings; these feelings do not come from the rational part, but from the emotional part. We can affirm that sympathy has more to do with the subjective expression of feelings and thoughts, while empathy seeks an objective understanding of the other’s inner world. This is seen in the presence of feelings of grief and sorrow for the death of the child, as well as in the physical expression of those feelings: crying.

*“... the parents when I talk to them are grateful and the fact that you cry with them because then they see that you have felt the same and that comforts them a little bit to feel that with you at that moment... it helps both of you to mourn.”*

*E4P1L21*

*“... the fact that I am a mother helps me to get closer to those parents... I try to get closer to them because I would like them to get closer to me if I were in the case...”*

*E10P2L2*

Crying with the family is understood by those interviewed as a sign of “good treatment” and “empathy” for the family, as you share the pain of that loss. This behaviour, although human, is still a sympathetic–affective behaviour, far from the concept of empathy.

### 3.3. Coping Strategies in the Face of the Death of the Child Admitted to the ICU

This is one of the core aspects of the research work.

Generally, the professionals adopt a dual role: they do not show the negative feelings emanating from the death of the patient they are caring for to others (especially to colleagues); distancing and emotional coldness appear as a practice in the clinical environment.

*“The nurse needs to draw enormous strength from within himself, he needs, at these moments, to adopt a feeling of coldness that often does not belong to him. Holding back the tears... it is not always possible, but it often becomes essential!”*

*E10P1L18*

*“It is important for nurses to know how to control their emotions so as not to harm or diminish their professional development.”*

*E9P1L11*

However, these feelings are present and appear when the professional is in a psychologically safe and calm environment (usually alone), such as at home or in the car. Crying is the most common behaviour.

*“... if I have to cry I cry… I prefer to cry at home... but when I am at work, I have to be there... and hold on like a champion... so I separate myself a little bit emotionally so as not to cry with them, for that child they have just lost... I am also a mother...”*

*E7P1L16*

*“After a bad day... where one of the children I care for dies... I sit and cry in the car, alone... before I get home... so that they don’t see me and worry. I like my job… but I don’t want others to think I don’t… because I cry when I come out of it and have a bad day.”*

*E6P2L5*

*“... I can’t talk about these feelings of sadness at home; nobody would understand them... or at least they would get tired of hearing them; nobody wants you to say that a one-year-old child died on your shift, after falling into a swimming pool and drowning, after more than 3 h of unsuccessful resuscitation. And that you’re the one who died... you swallow these things. It’s part of our job.”*

*E5P2L3*

*“... I deal with them (negative feelings) with another colleague, who I know has had a bad time, and I know she will understand me... I just want her to listen to me... and when I get it off my chest... I feel better.”*

*E8P2L6*

*“(What’s helps me... ?) … Verbalizing it without a doubt, saying it out loud seems to make it real, it helps to say it... we also know how to support each other in that sense, we generally do feel the support among ourselves.”*

*E6P2L9*

*“... I try to hide, when I get home... to have my children or my husband tells me things... things that distract me... those occupy my mind, and don’t make me think about the child I left in the hospital... I don’t want to be alone...”*

*E2P2L3*

*“.. I give hugs and kisses (more than usual) to all my family, especially my children and my husband... I am lucky that what happened to the family I just left in the hospital doesn’t happen to me.”*

*E4P2L16*

*“I do physical exercise... it’s a way of releasing tension and adrenaline... I clear my head by running or cycling... what I couldn’t do would be quiet things like reading a book or watching a sad movie.”*

*E7P2L6*

As can be seen, the behaviours are varied, especially characterized as avoidance behaviours: recounting feelings (without delving into them); psychological distraction behaviours from negative thinking (which remains); reaffirming family ties, without explaining the reason; and strenuous physical activity. All these behaviours try to mask the negative feeling derived from the death of the child, not to work on it.

In our research, nurses report that they do not know how to deal with negative feelings after experiencing the death of a child they care for. They do not know if it is “appropriate” to show their negative feelings in public in front of the family and if they can be seen as vulnerable and express themselves as they feel.

### 3.4. Lack of Training and Institutional Support

The interviewees recognize their limitations and lack of knowledge in dealing with these situations and negative feelings, and emphasize that everything they have been able to learn has not been based on training, but on personal and professional life experiences as described above.

*“... I don’t cry because I don’t know where my limit is as a professional... I don’t know if it is good for me to cry for my patient with his parents, or if that is not professional... if they had taught me how to manage it, I could tell you, look, if I can do this, I can shed a tear... but as we are not taught emotion management tools, each one learns how he/she can.”*

*E6P2L1*

*“... because we don’t have any training despite having been here... for many years, we don’t have any training on what to do well and at least we have concluded that there are many times what it is better not to do when you don’t know what to do.”*

*E4P2L2*

*“... it is true that here we have had to learn in fits and starts.”*

*E6P1L18*

*“... this experience has increased our knowledge, even if it’s self-taught, as they say, by beating ourselves up.”*

*E2P2L1*

*“It is notorious the lack of preparation of the professional to deal with death (...) This confrontation is always difficult because we are not prepared (...) although death is an event present every day or more frequently.”*

*E1P2L5*

*“I would appreciate the presence of a psychologist at the center to help me manage the knot in my stomach with which I have gone home, some days because I don’t know how to deal with the bad (...).”*

*E4P2L8*

*“I think that the psychologist is important on a general level... on a personal level, it is very important... on a personal level, it is very important... I think that tomorrow another child will come to me with the same circumstance and if I am still suffering because of a previous child... I won’t be able to do my job well... and then in the end you get into the same loop...”*

*E5P2L16*

*“Yes, it is important to have a person to help us with those negative feelings... and to deal with the families... and help us with all that.”*

*E9P2L1*

The interviewees indicate that psychological assistance to nursing staff would prevent decline in the care of subsequent patients and even in the development of their non-professional life; this service should be provided by the hospital centre as part of the institutional support and recognition of the professionals. However, the interviewees are unaware of the existence of a clinical psychology service in the centre, to which they can turn through official channels to resolve this type of aspect. The training offered by the Continuing Training Service of the Hospital does not include workshops on managing emotions or coping strategies for dealing with the death of a patient, which is a historical demand of the group.

## 4. Discussion

From the results of the interviews, the participants indicated that the death of a child they are caring for is a painful process for them and generates negative feelings, partly derived from a process of apparently misunderstood empathy. These feelings are not always confronted directly but are hidden, and they do not know to what extent these feelings can be expressed openly. Most of the coping strategies shown can be considered as avoidance strategies, since they try to face the negative event and the negative feelings derived from it, and to “forget” what they experienced, focusing on what gives them personal satisfaction and, for both, find temporary relief. Among them, in the testimonies collected, we find that the participants (a) discuss the case with colleagues, without going into the substance of the matter, and (b) reaffirm family ties or exercise. Finally, from the testimonies provided, it can be deduced that, in this PICU, there is no program for the reaffirmation of the personnel and psycho-affective reinforcement of the personnel after suffering a traumatic event such as the death of a child; the interviewees themselves indicate that they would like to have tools for the better management of the negative emotions that arise during work.

During the interview, when the participants were questioned about the way in which they cope with the death of a child, their responses were expressions of sadness and crying, testimonies of grief rather than strategies for coping with this grief, which conveys the idea of confusion between the two, when they are not the same thing. Grief is the expression of affliction or sadness derived from the feeling of loss, while coping is the mechanisms for coping with and combating this negative feeling. We can deduce from this situation that there is a complete lack of specific training, or that the conceptual and value map of the professionals involved needs to be further explored.

The use of crying as an expression of the sadness felt by the death of a patient is a common trait among nurses who care for dying or dying patients. However, it is not a coping strategy that alleviates the situation that the nurse is experiencing, or that improves as a strategy for the future. Even excessive expression of sadness, or getting carried away by the emotion of the moment, is negative at a professional level: the mental deterioration shown by the nurse can be detrimental to her ability to care for the rest of the patients she/he cares for [23].

The sadness and grief findings are not anomalous or unusual, in this context. Evidence from healthcare staff’s feelings in general and nurses’ in particular, regarding the death of a patient they are caring for, has been abundant for some time [24,25,26,27]. The literature describes emotional responses by nurses to the death of patients, such as disbelief, helplessness, loss, and guilt [28,29,30], feelings that correlate with the findings found in the participants of this research. This is a phenomenon that has not changed and around which significant progress has been made: the death of a patient generates negative feelings. This statement seems axiomatic in the light of the abundant evidence on the subject.

The most common individual coping mechanisms used by nurses in critical contexts before the death of pediatric patients [29,31,32,33] included strategies through support from other staff members, such as peer support, and refuge in individual spirituality. Peer support involves trying to share the negative experience with other people who have gone through the same thing, and understand their situation. This means that the topic is not discussed at home, or outside of the peer environment, whether within the clinical setting or outside. Another characteristic of this type of coping is the fact that this peer support is carried out in an “informal” way, far from the ordinary circuits of the healthcare center where they work [34,35,36,37].

Among those interviewees who identified specific behaviours for coping with the death of a child they cared for, we found behaviours such as “exercising” or “focusing on their family members and family activities”, behaviours that coincide with what several authors [37,38] defined as “distancing” and were defined as a form of distraction-oriented coping. In other words, by using this coping method, one tried to forget the painful event.

Some of the testimonies indicate a capacity for abstraction or the separation of events occurring in the clinical setting from their personal lives—even in the same work environment, the capacity to separate negative situations related to the death of a patient from the rest of the care actions. This is known as compartmentalization. It is identified as those behaviours that consciously or unconsciously separate what happened in the clinical environment from events outside it—to block the experience of the pediatric patient’s death from the rest of personal or professional actions [38].

However, the lack of coping strategies is also not a casual finding, which is not new to us. Frommelt [39] indicated that 76.5% of nurses surveyed felt inadequately prepared to care for the death of a patient. Despite the different strategies reported by interviewees, our participants expressed the need for more support and education/training to deal effectively with pediatric death, children’s families, and their own bereavement.

As we observed in our participants, the learning of these coping strategies mainly occurs, in most cases, from one’s own professional experience, and from the observation of the behaviours of other colleagues. Unfortunately, there are no teaching experiences that show the effectiveness of providing stress coping strategies [40].

There is minimal education to provide sufficient care to dying patients and their families in these difficult situations. There is abundant evidence indicating that the absence of coping strategies is already present even in newly graduated nurses, indicating systemic structural defects in the training of professionals, where the training of nurses on death and dying in many undergraduate nursing programs often leaves the students unprepared for the phenomenon of a patient’s death—coping with the patient’s own death, the family, and their own feelings about it [11,30,31].

Institutions such as the American Association of Colleges of Nursing created the End-of-Life Nursing Education Consortium (ELNEC) as a way to improve palliative care education initiatives [41]. This programme encourages the incorporation of end-of-life care into undergraduate and postgraduate education in the areas of particular bereavement issues, such as palliative care, critical care, and pediatric nurse specialists, as well as nurse educators responsible for the education of future nursing professionals.

The experiences of new nurses who received ELNEC education in their nursing programs and who cared for dying patients during their first year of practice indicate that they had useful insight to meet the challenges of implementing dying care in clinical practice, as well as to face this task without suffering for them [34].

Simulation-based training programmes to work on nursing competence in relation to coping with the death of a patient have demonstrated their efficacy, especially in the communicative aspects towards the patient and family [42,43,44,45,46], and are suggested in comparison to traditional training programmes.

Gillman et al. [47] found that effective strategies included those that: (a) foster connections within the team; (b) provide education and training to develop behaviours that help control or limit the intensity of stress or aid recovery; and (c) help process emotions and learn from experiences. While individuals must take responsibility for developing personal strategies to help with coping and resilience, organizational support is integral to equipping people to deal with work-related challenges.

As the evidence indicates, the strategies displayed by the participants in our study can be framed within emotion-focused coping strategies, such as distancing, avoidance, delusion, and positive reappraisal. These strategies focus on reducing or managing the emotional distress resulting from the crisis and do not seek to alter the external environment. As a counterpoint, or in a complementary way, we find the so-called problem-focused strategies. These aim to solve the problem or take action to change the source of stress and are usually invoked when constructive action can be taken, whereas emotion-focused coping is used when people feel that the situation cannot change and must be endured [48].

The effectiveness of any particular coping strategy varies according to the situation, and there is not one generally accepted way for adults to cope with stress.

Another aspect to be analyzed is the presence of expressions related to desires to abandon the profession or vocational questioning after the death of a child, especially when this occurs in “traumatic” circumstances on a social level, and is not expected. These ideas relate to the concept of compassion fatigue (CF). This is typically understood as emotional, physical, and spiritual exhaustion from “witnessing and absorbing the problems and suffering of others” when working with traumatized individuals [49]. This negative feeling, generated by the continuous and unfulfilled confrontation with the suffering of others, is experienced first-hand by intensive care nurses caring for those individuals or families who are suffering, both adult [50] and pediatric patients [51,52]. CF, according to Figley [53], is “the cost of caring”, and occurs in the absence of mechanisms to identify and manage the emotional residue of this contact.

CF is currently a major focus of study worldwide, especially in critical care and emergency departments, with prevalence rates ranging from 30–40% of professionals in these units [54,55], and slightly lower in PICUs (25%).

CF is usually related to phenomena such as post-traumatic stress disorder and burnout, all of which are related to the desire of nurses in highly demanding units to leave. Therefore, detecting this problem in professionals is a necessary task for the personnel managers of the units, as well as promoting activities to prevent it. This suggests the need to explore this factor in more detail in future studies, due to the high incidence of this issue.

Mental models are a person’s deeply ingrained images of how the world they live in works, images that limit us to familiar ways of thinking and acting. We are often unaware of our mental models and the effects they have on our behaviour [56].

The mental models of individuals working in a common space, such as a hospital unit, blend to form collective assumptions, generalizations, and images, which influence their behaviour and perceptions of their realities. These shared paradigms, as with the mental models on which they are based, are tacit (i.e., they do not need to be made explicit at any point) [57]. In this way, the mental model of one person, immersed in an organization, is diluted and reaffirmed in the actions of all other colleagues.

One of the mental models that transcend the testimonies in an interpretative way derives from the fact of feeling “grief” for the death of a child; this negative feeling is naturalized, derived from an erroneous concept of “empathy”. Thus, the good nurse is the one who shows tears and grief together with the relatives, as a sign of “accompaniment”.

In this sense, empathy, in the clinical context, differs from sympathy in that its main emphasis is not on the perception of the emotional charge that accompanies human relationships, but on the eminently cognitive component that requires understanding the concerns of the patient and their family, and also knowing how to incorporate this knowledge into communication with the patient during treatment [58]. For empathy, as a professional competence, emotions are important, but even more important is the control one has over them in the management of the relationship with the patient.

Therefore, we understand that empathy implies enhancing the cognitive dimension, while sympathy enhances the emotional dimension. The implications of this difference are important considering that, in the case of nursing professions, the emotional charge that accompanies dealing with patients can be detrimental to both them and their patients if it gets out of control [58].

There is a broad consensus among health science researchers and educators on the importance of empathy development in the training of health professionals. With regard to the existing training gap, despite the paucity of studies and the variability of measures across studies, the evidence shows training gaps in undergraduate states in relation to the acquisition of empathy [58]. Again, clinical simulation is shown to be an effective training strategy for this complex competency compared to traditional training methods.

However, the concept of empathy needs to be properly conceptualized in clinical reality and may be insufficient [59]. Studies in clinical settings have shown that the development of an emotion-focused empathic relationship can be detrimental to patients and the professionals responsible for their care [59,60].

## 5. Limitations

This study was conducted in a single PICU of a public hospital in the southeast of Spain. Therefore, the findings of this study may not be the same as those found in other hospitals in the country. That is why studies based on mixed-type methodologies, with the inclusion of a representative sample from other centers, are necessary to reaffirm the findings, or to consider them as a local phenomenon. However, the correlation of the findings found, with available evidence, can inform PICU nurses about the grief phenomena that occur.

## 6. Conclusions

This study provided insight into the experiences, emotional responses, and coping strategies of pediatric nursing staff who work in a Pediatric Intensive Care Unit, when they confront a death of a child they are caring for.

The nurses conveyed messages based on negative personal emotions such as helplessness and grief.

These nurses sought their way of coping with grief by sharing with colleagues, exercising, and focusing on family members and family activities, which can be understood as emotion-focused coping strategies. These strategies were learned through their own experience and the values of the institution itself. A wrong mental model around the concepts of empathy/sympathy can be deduced from the testimonies collected, which makes the staff become emotionally involved with the pain of the families—pain that they do not manage adequately. There is a high risk of compassion fatigue, derived from the use of inadequate coping strategies, so a detailed analysis of this phenomenon is necessary, as well as a structured training program in coping strategies and institutional support for the professionals in this unit.

The results of this study can help the management, nursing managers, nursing educators, and healthcare staff to idealize and promote collective actions of confrontation and activities that strengthen the union of the multi-professional team through continuing education.

It is necessary to create spaces where the individual and collective expression of problems and thoughts is valued, consolidating the bond between management, supervisors, and workers, so that they feel supported in times of weakness, and, from this, new forms of individual and collective coping can be built, making the worker the protagonist of the construction of their personal well-being.

## Figures and Tables

**Table 1:** Information about participants.

Nickname	Gender	Age (Years)	Married/Children	Nurse Experience (Years)	PICU Experience (Years)	ICU Adult Experience (Years)
E1	Female	22	No/No	2	1	No
E2	Female	24	Yes/No	2	1	No
E3	Female	31	Yes/Yes	8	8	No
E4	Female	36	Yes/Yes	12	12	No
E5	Female	36	Yes/Yes	13	13	No
E6	Female	37	Yes/Yes	14	14	No
E7	Female	42	Yes/Yes	21	18	Yes
E8	Female	42	Yes/Yes	21	15	Yes
E9	Female	43	Yes/Yes	22	16	Yes
E10	Male	47	Yes/Yes	25	22	No

## Data Availability

The data are available upon email request to the corresponding authors.
